# Novel hydrogel poly (GG-*co*-acrylic acid) for the sorptive removal of the color Rhodamine-B from contaminated water

**DOI:** 10.1016/j.heliyon.2023.e19780

**Published:** 2023-09-03

**Authors:** Salma Jabeen, Sultan Alam, Luqman Ali Shah, Muhammad Zahoor, Muhammad Naveed Umar, Riaz Ullah

**Affiliations:** a Department of Chemistry, University of Malakand, Chakdara Dir Lower, KPK, 18800, Pakistan; b National Center of Excellence in Physical Chemistry (NCE), University of Peshawar, Pakistan; c Department of Biochemistry, University of Malakand, Chakdara Dir Lower, KPK, 18800, Pakistan; d Department of Chemistry, University of Liverpool, UK; e Department of Pharmacognosy, College of Pharmacy, King Saud University, Riyadh 11451, Saudi Arabia

**Keywords:** Hydrogel, Rhodamine B, Adsorption, Kinetics

## Abstract

Textile effluent's treatment is highly desired due to the presence of hazardous, water-soluble and non-biodegradable dyes that not only have harmful effect on the environment but on living beings as well. Treatment of these pollutants by sorption through biosorbents is considered to be a best method of choice due to greener nature of the processes. In this connection hydrogel sorbents might be an intriguing option due to its straightforward application, great efficacy, easy synthesis, rapid turnaround, and potential of recycling. Herein, novel hydrogel was prepared using Gellan Gum and acrylic acid (GG-co-AAc) which were then characterized by instrumental techniques like UV/visible and FTIR spectroscopy, SEM, EDX and XRD. The anionic hydrogel's adsorption capacity, swelling behavior, and sorption potential were determined using Rhodamine-B as potential environmental pollutant. The hydrogel exhibited an impressive adsorption capacity of 1250 mg/g. Swelling experiments were performed in Milli-Q distilled water at different pH levels, reaching maximum swelling of 3230% after 23 h as determined through Fickian diffusion. At pH 7, the anionic hydrogel's sorption potential was thoroughly studied in the subsequent experiments. The adsorption process was found to follow the Langmuir isotherm, indicating a monolayer adsorption mechanism supported by higher R^2^ values compared to the Freundlich isotherm. Thermodynamic analysis revealed the exothermic nature of the adsorption process, with a negative enthalpy value of −11371 KJmol^-1^ and negative entropy value of −26.39 Jmol^−1^K^−1^, suggesting a less ordered system. These findings provide valuable insights into the adsorption characteristics and potential applications of the synthesized anionic hydrogel.

## 1 Introduction

The textile, leather, cosmetic, pharmaceutical, and paint industries generate significant amounts of byproducts and waste dyes, posing significant threats on environment [[1], [2], [3], [4]]. These industries commonly utilize water-soluble, toxic, and non-biodegradable dyes. When released into water bodies, these dyes cause undesirable color changes, reducing the transparency of the water. Consequently, sunlight penetration is hindered, affecting oxidation and photosynthetic activities in water reservoirs [5]. Water is a vital resource essential for the survival of all life forms on Earth. It plays a direct role in sustaining various ecosystems and supporting human well-being. Unfortunately, the rapid progress in living standards, industrial development, and population growth has put immense pressure on water bodies. According to a report of the United Nations, over 2 billion people around the globe currently lack access to clean and safe water, and this number is projected to increase significantly, to nearly 1.8 billion people that would be expected to face water scarcity by 2025 [6]. Due to rapid industrialization, water has become a sink of these toxins, which move up through the food chain with progressively larger quantities than the organisms that came before until they reached humans [7].

In humans, the cationic dyes interact with the anionic surface of the plasma membrane and go into the cytoplasm of the cell, where they concentrate and causes various health complications [8,9]. Cancer, tissue necrosis, jaundice, stupor, eye irritation, and diarrhea are only a few of the severe health problems that people experience when cationic dyes accumulate in their tissues [10,11].Synthetic dyes have become a favored choice in industries and art due to their vibrant, long-lasting colors surpassing natural dyes. This breakthrough has elevated design possibilities, meeting consumer demands with higher color intensity and fastness. Their allure lies in enhancing visual impact across diverse domains, revolutionizing numerous fields with more efficient chromogens [12].

The sorptive treatment of dye-containing wastewater is an attractive approach, even with the availability of several conventional and cutting-edge methodologies [[13], [14], [15]]. The method of using hydrogel sorbents, composed of extensively cross-linked 3D-arranged hydrophilic polymer chains, is favored for its ease of use, high efficiency, rapid recovery, and potential for multiple reuses [16]. These hydrogels have significant swelling capacity in water, physiological fluids, and saline solutions [11], leading to diverse applications in catalysis [16,17], medicine [18], wastewater treatment [16,19], and agriculture [17,18]. Their attractiveness for research lies in their high sorption capacity, ease of recovery, and potential for reuse [20,21]. Numerous organic hydrogels, such as polyacrylic acid (p(AAc)) [22] and poly (acrylic acid-*co*-gellan gum) (p(AA-co-GG)) [23], have been created for diverse industrial applications. Among them, the pullulan/kaolin hydrogel nanocomposite (f-PKHN) was developed as a highly effective adsorbent (referenced from Ref. [24]) for eliminating paracetamol (PCT), a pharmaceutical pollutant, from liquid-phase contamination.

### 1.1 Top of form

The study of gellan gum-based hybrid materials for dye removal from wastewater has gained considerable attention due to their promising adsorption properties, cost-effectiveness, abundance in nature, and eco-friendly nature. Bentonite and alginates have emerged as potential effective sorbents when combined with gellan gum, offering the advantage of synergistically combining adsorption capabilities with high surface area, porosity, and ion exchange capacity. These hybrid systems hold great promise for the development of efficient and sustainable approaches in dye removal for wastewater treatment [25,26]. Gellan gum-based hybrids with bentonite and alginates are eco-friendly and cost-effective for dye removal [27,28]. Various hydrogel compositions, including poly(AAc) [29,30], polyGG [31], poly(GG-*co*-AAm-AAc) [32], GG-cl-poly(AAc-*co*-MAAc) [33], and guar gum-co-acrylic acid [34], have shown promise in sustainable wastewater treatment.

### 1.2 Top of form

Gellan gum-based hydrogels (GG-cl-poly(AA)) were synthesized using a free radical graft copolymerization technique with acrylic acid as a monomer, N,N-methylene-bis-(acrylamide) as a crosslinker, and ammonium persulfate as an initiator. Response surface methodology (RSM) and central composite design were employed to optimize the synthesis process and obtain the highest percentage swelling. The optimized hydrogel demonstrated enhanced water retention capabilities, making it suitable for moisture stress agriculture. Additionally, GG-cl-poly(AA) exhibited significant biodegradation behavior and environmentally friendly characteristics, indicating its potential for cost-effective and sustainable agricultural applications [35]. The synthesized poly(gellan gum-co-acrylamide-co-acrylic acid) (GGDA) hydrogel with improved structure, thermal stability, and porosity compared to gellan gum demonstrates excellent adsorption abilities for methylene blue, following a pseudo-second-order kinetic model and Freundlich isotherm. The pH-sensitive GGDA hydrogel exhibits a spontaneous, exothermic adsorption process with a maximum adsorption capacity of 423.46 ± 13.60 mg/g, showing potential as an effective alternative adsorbent for organic dye removal from aqueous solutions [36].

The incorporation of gellan gum into acrylic acid enhances the hydrogel's properties, including swelling behavior, sorption capacity, mechanical strength, and biodegradability, while reducing cost and toxicity. The novel poly(gellan gum-co-acrylic acid) composite hydrogel, synthesized via free radical polymerization, exhibits promising sorption capacity for Rhodamine B at pH 7 and aims to contribute to the understanding of organic-inorganic hydrogels.

## 2 Experimental

### 2.1 Materials

We used products from Sigma Aldrich, including acrylic acid (AA), gellan gum (GG), N,N-methylene bis acrylamide (MBA), tetramethyl ethylenediamine (TEMED), and ammonium per sulfate (APS).The analytical grade Rhodamine-B adsorbate from Sigma Aldrich is also known as RhB (purity 99%). The *Sphingomonas elodea*, which has been shown to flourish on elodea plants, is used to make gellan gum (GG) (formerly known as pseudomonas elodea). The chemicals were used straight from the source in their original form since they were of analytical quality. The chemicals were used straight from the source in their original form since they were of analytical quality. Additionally, the sorption studies that followed the solution's creation employed Milli-Q ultrapure water with a resistivity of 18.2 M cm at 25 °C.

### 2.2 Fabrication

**Scheme 1:**
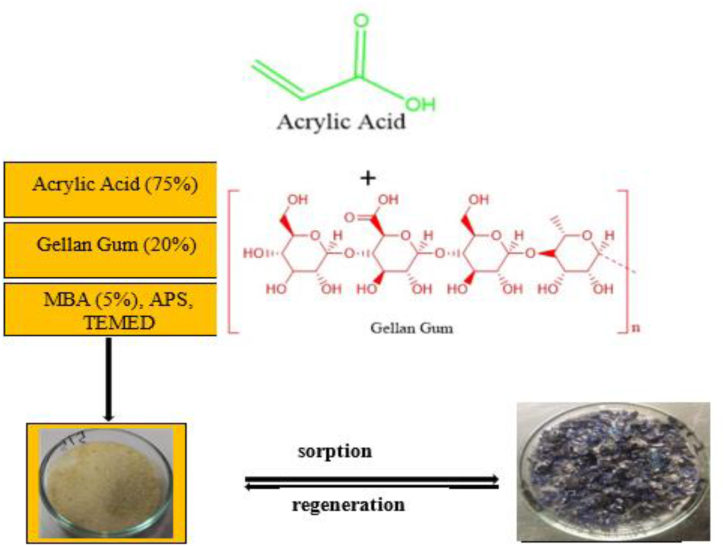
Schematic presentation of the hydrogel preparation.

Using in situ free radical polymerization, the co-polymeric poly(Gellan Gum-*co*-Acrylic acid) hydrogel, also known as p(GG-*co*-AAc), was created. Briefly stated, Milli-Q deionized water (MQ-DW) was carefully charged with 1 ml AAc (85%), 1 g GG (20%), and 0.061 g MBA (5%) (Scheme 1). About 0.05 g APS and 20 μL TEMED were added after the chemicals were make it easier for users and sonicated at 298 275K to homogenize them in MQ-DW. Argon purging was used to eliminate any oxygen from the resulting solution that may have gotten in the way of the intended reactions. The co-polymeric poly(Gellan Gum-*co*-Acrylic acid) hydrogel, usually referred as p(GG-*co*-AAc), was produced via in situ free radical polymerization. Briefly stated, 1 ml AAc (85%), 1 g GG (20%), and 0.061 g MBA (5%) were carefully charged into Milli-Q deionized water (MQ-DW) (Scheme 1). Just after chemicals were give the best and sonicated at 298 ± 275K to homogenize them in MQ-DW, 0.05 g APS and 20 μL TEMED were added. Any oxygen in the final solution that would have interfered with the planned reactions was removed using argon purging.

### 2.3 Characterization

SEM was used to view the texture of the p(GG-co-AAc) hybridized hydrogel at different resolutions (JSM-5910, JEOL). Shimadzhu1800 UV–vis & PerkinElmer spectrum-10.5.1 FTIR spectrophotometers were used to measure the hybrid material's spectroscopic response in the UV–vis (200–800 nm) and FTIR (4000-400 cm^−1^) wavelength ranges, respectively. Energy - dispersive x X-ray (EDX) and X-ray diffraction (XRD) techniques were used to determine the elemental composition and crystallinity of the hydrogel (JDX-3532, Make: JEOL, Japan).

### 2.4 Swelling study

Gravimetric measurements were made of the hybrid hydrogel's percent swelling study throughout a wide pH range at room temperature (25 ± 2 °C) (4–11). The hydrogel composite was first carefully cut into a tiny block measuring 1 cm^2^ and dried thoroughly at room temperature in an electric oven. The dried block was weighed accurately, dipped in Milli Q water, and then removed and weighed after a set amount of time. After noticing the weight gain, Eq (1) continued to calculate the swelling percentage

(1) PercentSwelling=(ms–md)/md×100

The formula for percent swelling is as follows: ms = mass of the swollen components of the hybrid hydrogel, and md = mass of the dry components.

### 2.5 Sorption experiments

Using a widely used batch adsorption technique, the hybrid hydrogel's sorptive potency against RhB was examined. Protocol required that 10 mL of the requisite RhB concentration and 0.01 g of hydrogel be added to a reagent bottle in turn for the sorptive treatment (RB). The pH of the slurry then was carefully adjusted to the required value using a 0.1 M aqueous solution of either NaOH or HCl. The Round Bottles (RB) was shaken ferociously (at 200 rpm) for the specified amounts of time in a thermostated steam bath (TSW) that was preheated to the proper temperature. The remaining RhB substance in the slurry was subsequently filtered, and its photometric detection was carried out using a UV–vis spectrophotometer. In the absence of hydrogel sorbents, additional studies on aqueous RhB in RB showed that there was negligible dye retention on the container walls. To guarantee accuracy, the sorption tests were performed three times. Graphs and tables were then made using the average value of the tirade.

#### 2.5.1 Sorption kinetics

At various temperatures (20, 40, and 60 °C) and for five to 60 min, the sorption kinetics of RhB over p(GG-co-AAc) hydrogel were examined. Each of the six round bottles (RBs) was first given 10 mL of an aqueous Integration of sustainability and 0.01g of a superabsorbent in a series of additions. The RBs were then immersed in a TSW bath and violently shaken at intervals of 10, 20, 30, 40, 50, and 60 min at 200 rpm. According to Scheme 1, p(GG-co-AAc) hydrogel is produced in a single pot at 60 °C.

#### 2.5.2 Sorption isotherm

Similar to sorption kinetics, each RB was charged with 0.01 g of precisely weighed sorbent after receiving 10 mL of a freshwater RhB solution. Each RB received an addition of solutions with initial concentrations ranging from 0.0005 to 0.003 M. Then, each RB's mixes were warmed to a pH of 7, added to a TSW-bath, and vigorously stirred for in an hour at 30 °C utilizing 200 rpm.

## 3 Results and discussion

### 3.1 Characterization

#### 3.1.1 Surface morphology

**Fig. 1:**
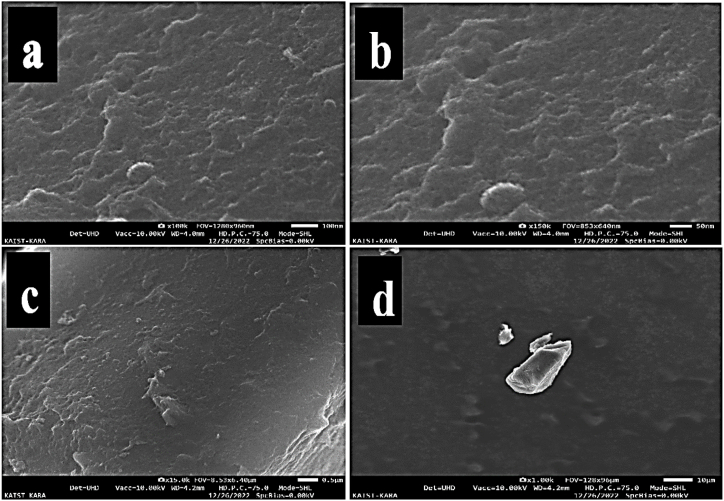
SEM images of p(GG-co-AAc) hydrogel captured at (a) 100 nm (b) 50 nm (c) 0.5 μm and (d) 10 μm respectively.

The successful creation of the unique p(GG-co-AAc) hydrogel was demonstrated, and SEM images were used to establish its rough surface shape (Fig. 1 a to d). Grafting alters the hydrogel's size and surface, resulting in morphological changes. Their morphology has considerably altered. The GG's surface has fractures all throughout and appears fibrous. The formation of covalent links between diverse polymer chains during crosslinking makes GG-co-AAm appear smoother surface. A favorable runner for dye adsorption in aqueous media is provided by the hydrogel's behavior because the both material used for hydrogel polymer have a hydrophilic surface, which mean they have an affinity for water adsorption. They polymer chains of gellan gum have hydrophilic functional groups, which can interact with water through hydrogen bonding. This makes the polymer an effective for dyes adsorption. They produced microparticles had an amorphous shape and a smooth yet deformed surface morphology. The size of the particles varied from 9.38 to 10.67 m [37,38]. Similar outcomes, including micrographs of various hydrogels with Gellan gum and acrylic acid included, have also been reported elsewhere [39,40].

#### 3.1.2 FTIR of the hydrogel

**Fig. 2:**
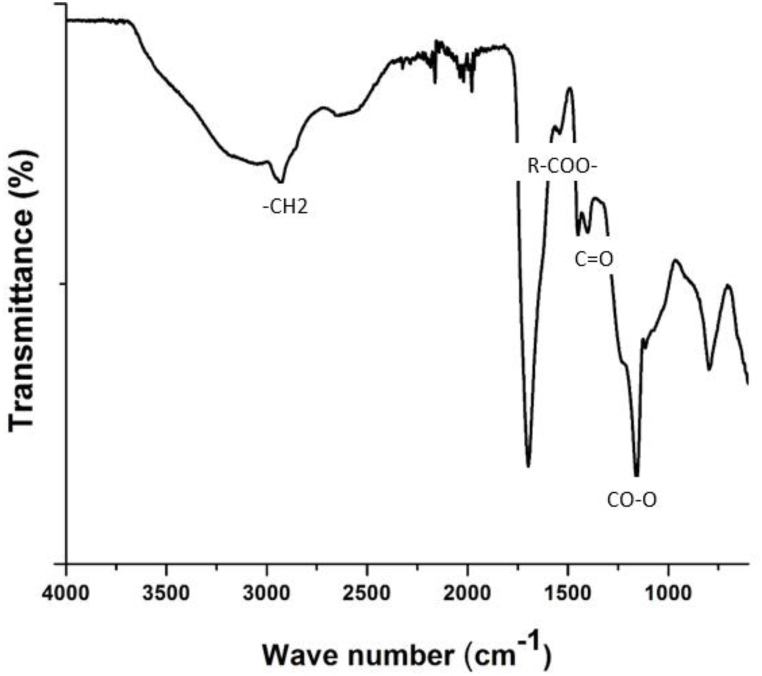
FTIR spectra of p(GG-co-AAc) hydrogel.

Stretching of the -*O*-H and carbonyl (C=O) functionalities of the AAc segment results in intense (1712 cm^−1^) and broad (3547 cm^−1^) signals in the copolymeric p(GG-*co*-AAc) hydrogel's FTIR spectrum (Fig. 2) [[41], [42], [43], [44]]. The development of bands at 1448 cm^−1^ and 1541 cm^−1^, attributed to the symmetric and asymmetric stretching of the C=O group, respectively, provided additional proof that AAc was present in the hydrogel matrix. The resultant signal (1448 cm^−1^) may be an overlapped response of C=O symmetric stretching and –CH2- vibrational bending in copolymeric superabsorbent because the distinctive bending vibration of methylene (-CH2-) may also give absorption band in this area. The peak at 1165 cm^−1^ may also attest to the incorporation of AAc in the hydrogel because the signal is derived from stretching vibrations of the CO–*O*- moiety found in the acrylate ion created by AAc ionization. The assignment bands at 1627.81 and 1409.87 cm^−1^, which are caused by the asymmetric and symmetric stretching of the carboxylate group, were used to establish the presence of the second monomer, GG, in the hydrogel matrix. While the bands at 1153.35 and 1024.13 cm^−1^ are caused by ethereal and hydroxylic C–O stretching's, respectively, the band at 2927.74 cm^−1^ is caused by the stretching vibrations of the -CH2 group. The stretching vibration of the –OH group in the gellan gum hydrogel is indicated by the bending vibration of C–H that was seen at 3411 cm^−1^ for gellan gum. The existence of peaks for all hydrogel monomers in the FTIR spectrum confirms that the hydrogel that was attempted to be prepared using a free radical polymerization reaction in this research was successfully fabricated [[45], [46], [47], [48], [49], [50]].

#### 3.1.3 X-ray diffraction (XRD)

**Fig. 3:**
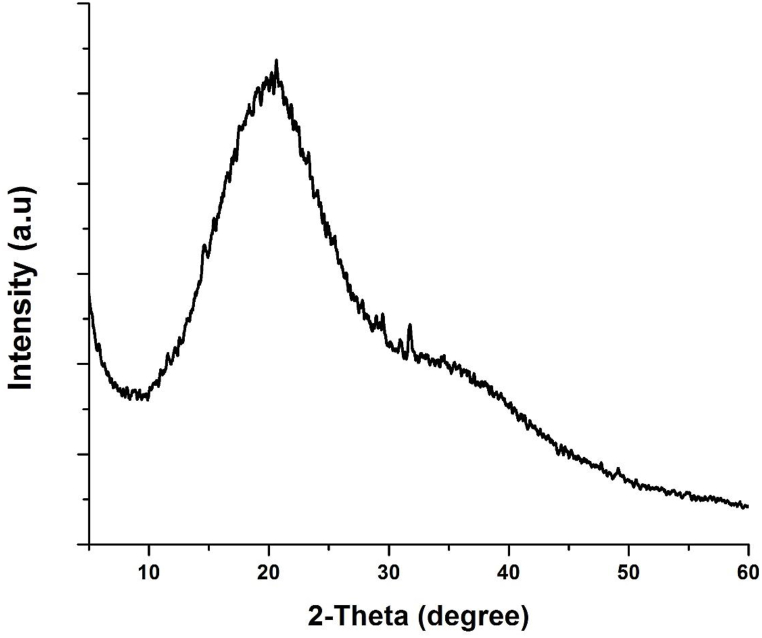
XRD pattern of (GG-co-AAc) hydrogel.

The XRD patterns of GG and AAc is shown in Fig. 3. Gum gellan is distinguished by a broad peak at 2.19°. The GG's main amorphous feature is indicated by its lower intensity diffraction pattern. But unlike gum gellan, the GG-co-AAc diffraction pattern showed the development of a cross-linked hydrogel network [51]. Two significant characteristic crystalline peaks are produced by the GG-co-AAc at 2° and 2°26°, respectively. There is also a peak at 28°, indicating that GG contains some crystals [52]. The XRD peak for GG-co-AAc moved toward a higher angle, strengthened, and widened. This shows that after grafting with AAc, GG-co-AAc converged toward a more orderly arrangement [[53], [54], [55], [56]]. Therefore, it strengthens the case for crosslinking in gellan gum [25].

#### 3.1.4 Energy dispersive x-ray (EDX)

**Fig. 4:**
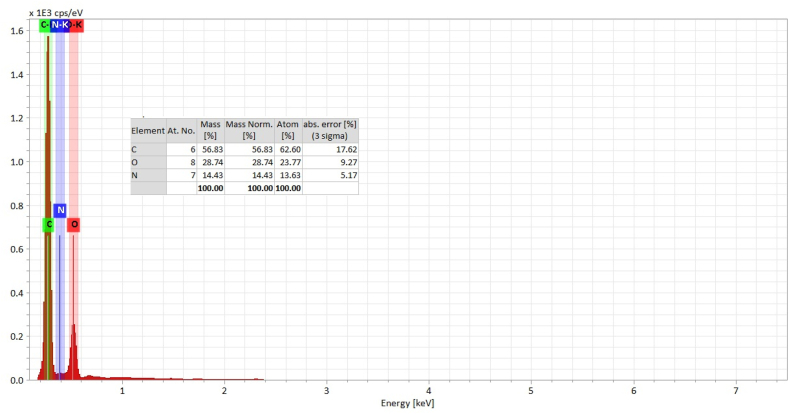
EDX spectra of (GG-co-AAc) hydrogel.

The EDX spectrum reveals that the hydrogel consisted only C, O, N which are presented in Fig. 4.

### 3.2 Swelling measurements

**Fig. 5:**
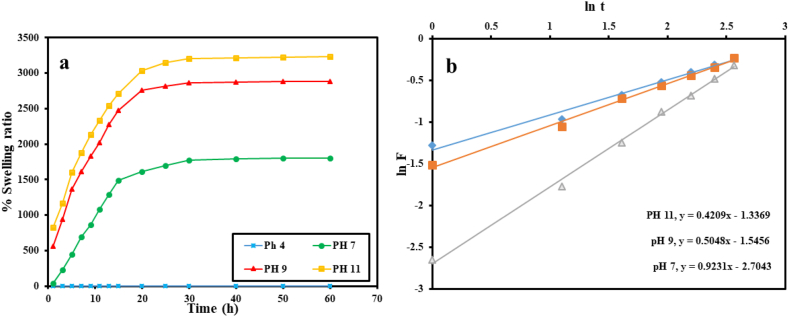
Percent swelling for p(GG-co-AAc) hydrogel at various pH levels (a) and ln F vs ln t plots (b).

The water absorptivity of the hybrid hydrogel exhibits an initial sharp surge, followed by a slow drop, which achieves equilibrium in 23 h, as measured by the % swelling over time in the pH range of 4–11. (Fig. 5a). The exceptional ability of the composite hydrogel to expand (3230% at pH 11) may be due to the antagonistic interactions between the carboxylate (COO) moieties found in AAc [57]. In addition, as pH rises, electrostatic repulsion increases, widening the pores and allowing water to more easily infiltrate into the composite hydrogel. The hybrid hydrogel exists in neutral form in an acidic (pH 4.5) environment due to significant hydrogen bonding that causes the skeleton to collapse, resulting in nearly closed pores and difficult water diffusion. Fick's law was used to examine the sort of water diffusion into the hybrid material during swelling [58]. Applying Eq. (2) to the swelling data allowed for the computation of the swelling fraction (F), which was then fitted into the following Eq. (3);

(2) F = St So = ktn

(3) lnF = lnk + nlnt

where St and So stand for balance swelling and % swelling at time t, respectively. Additionally, the constants k and n, respectively, stand in for the core network of the hybrid composite and the exponential distribution of the solvent. The slope and direction of the ln F against ln t plots in (Fig. 5b) were used to determine the n and k values (Table 1).

**Table 1:** Swelling of p(GG-co-AAc) hydrogel at different pH.

pH	n	K	R^2^
7	0.92	0.066	0.99
9	0.50	0.21	0.99
11	042	0.26	0.98

The plots were made using information from the first 10 h of the swelling testing, when the hybrid hydrogel absorbed 60% of the water (by mass). At all pH levels, the n values of the hydrogels are all smaller than 0.2, demonstrating Fickian water diffusion into the superabsorbent [59].

### 3.3 Proposed mechanism for the synthesis of GG-co-AAc hydrogel

The synthesis of gellan gum and acrylic acid hydrogel by free radical polymerization using APS (ammonium persulfate) and a cross-linker can be represented by the following mechanism:

**Formation of free radicals:** Ammonium persulfate (APS) is used as an initiator in this reaction. APS dissociates into two sulfate radicals, which are highly reactive free radicals. This reaction can be represented as follows:

(NH_4_)_2_S_2_O_8_ → 2NH_4_^+^ + 2SO_4_^•^

**Initiation:** The sulfate radicals attack the double bond of acrylic acid, initiating the polymerization reaction. This leads to the formation of a new free radical on the acrylic acid monomer.

SO_4_• + CH_2_=CHCOOH → CH_2_•-CHCOOH

**Propagation:** The new free radical on the acrylic acid monomer attacks another monomer, creating a chain reaction. This process repeats itself until the chain reaches a certain length.

CH_2_•-CHCOOH + CH_2_=CHCOOH → (CH_2_–CHCOOH)_2_•

**Cross-linking:** To form a hydrogel, a cross-linker is added to the reaction mixture. The cross-linker contains multiple functional groups that can react with the free radicals on the polymer chains, forming covalent bonds between the chains. This process is called cross-linking, and it leads to the formation of a three-dimensional network, resulting in the hydrogel.

**Termination:** The polymerization reaction is terminated by the combination of two free radicals or by the reaction with a chain-transfer agent.

Overall reaction:

(CH_2_=CHCOOH)n + Cross-linker → Gellan Gum and Acrylic acid Hydrogel

The gellan gum is produced due to the presence of the gellan gum molecule in the reaction mixture. Gellan gum is a polysaccharide that is commonly used in hydrogel synthesis as a rheology modifier. The gellan gum molecules interact with the polymer chains and the cross-linker, contributing to the final structure and properties of the hydrogel.

The specific cross-linker used in the reaction can vary depending on the specific application and desired properties of the hydrogel. The structure of the cross-linker depends on the type of functional groups it contains and the number of functional groups. The cross-linker can contain two or more functional groups that can react with the free radicals on the polymer chains to form covalent bonds.

Overall, the mechanism for the synthesis of gellan gum and acrylic acid hydrogel by free radical polymerization using APS and a cross-linker involves the formation of a polymer chain through the polymerization of acrylic acid monomers, followed by cross-linking of the polymer chains to form a hydrogel. The gellan gum molecule in the reaction mixture contributes to the final structure and properties of the hydrogel.

### 3.4 Sorption experiments

#### 3.4.1 Kinetics

For rate determination (which demonstrates sorbent effectiveness) and sorption process mechanism, the kinetics analysis of the sorption process is essential. Due to the abundance of sorption sites, kinetic studies carried out at various temperatures show an initial fast sorption rate. The sorption rate considerably decreased after 10 min because of RhB's superabsorbent slow absorption and the decline in the number of adsorbent surfaces [60]. To better understand the sorption kinetics of RhB, the following kinetic information was introduced to the pseudo first order (Eq. (4)), pseudo second order (Eq. (5)), and based on inter diffusion model (Eq. (6)).

(4) ln(qe − qt) = lnqe − k_1_t

(5) t qt = 1 qe t + 1 k_2_qe

(6) qt = Kdiff t 0.5 + C

**Fig. 6:**
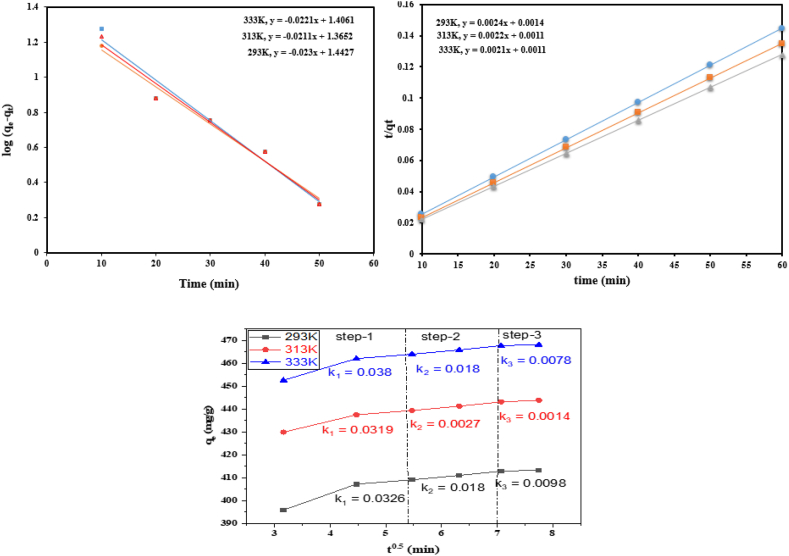
RhB sorption by hydrogel composed of p(GG-co-AAc): models for intraparticle diffusion, pseudo 1st order kinetics, and pseudo 2nd order kinetics.

The intercept and trend of the ln (qe-qt) against t plots shown in Fig. 6a were used to compute the kinetic parameters qe and k1, which are shown in Table 2 at various temperatures. The intercept and slope of the graphs in Fig. 6b were used to get the coefficients of k^2^ and qe for the pseudo-second order reaction as displayed in Table 2 at three various temperatures. The best data fitting into the pseudo second order model is clearly shown by the significantly higher R^2^ values of the pseudo second tool that generates to the pseudo first order graphs. The excellent agreement between theoretical and experimental qe values determined using the same model served as additional evidence for the validity of pseudo-second order kinetics for RhB sorption. On the other hand, the large discrepancy between the theoretically and experimentally estimated qe values using pseudo-first order equations suggests that the same model does not well suit the facts. Understanding the sorption process is necessary to determine the rate-limiting phase as well as the ideal sorption conditions. Fig. 6c depicts the t^1/2^ vs qt [61] plots. The early diffusion of RhB into the superabsorbent macropores is the fastest phase. The following, somewhat sluggish stage involves the dye diffusing into the mesopores. The diffusion of RhB into the hydrogel's micropores occurs in the third stage, which takes the longest [62].In similar fashion a study has been reported where the kinetics effects of lead and cadmium concentration over time has been investigated to determine the adsorption rate for the used adsorbent polyacrylamide/bentonite hydrogel nanocomposite. Batch experiments were likely conducted, where metal ions were exposed to the nanocomposite, and samples were taken periodically to measure remaining concentrations. Analysis of the data helped in determining the adsorption rate and potentially identified the most suitable mechanism or model for describing the process [63].

**Table 2:** Measured kinetic values for the sorption of RhB from several applicable models by p(GG-co-AAc) hydrogel.

Pseudo First Order				Pseudo Second Order			
**K**_**1**_**(min**^**−**^**^1^)**	qe (mgg^−1^) (cal)	qe (mgg^−1^) (exp)	R^2^	K_2_(mgg^−1^) (min^−1^)	qe (mgg^−1^) (cal)	qe (mgg^−1^) (exp)	R^2^
**0.052**	27.72	414.77	0.9668	8.066 × 10^−9^	416.6	414.77	1
**0.048**	23.18	445.01	0.9789	5.42 × 10^−9^	450.4	445.01	1
**0.051**	25.47	469.57	0.9737	4.89 × 10^−9^	474.1	469.57	1

#### 3.4.2 Sorption isotherms

**Fig. 7:**
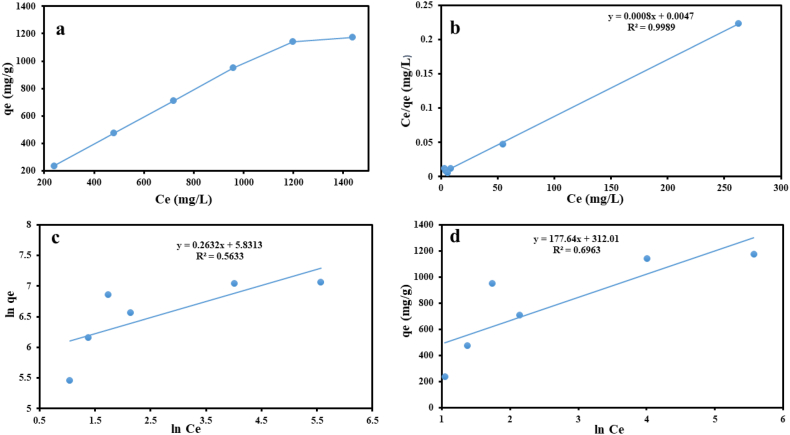
The variation of $C_{e}$ verses $q_{e}$ (a) Langmuir plot of $\frac{C_{e}}{q_{e}}$ verses $C_{e}\text{,}$ (b) Freundlich plot of ln$q_{e}$ verses ln $C_{e}$ Ce, (c) and Temkin plot of $q_{e}$ verses $C_{e}$ (d) for the sorption of RhB over p(GG-co-AAc) hydrogel at 293 ± 275 K.

It is essential to analyze the sorption isotherm in order to establish the maximum sorption limit when developing a column or batch system for the treatment of waste water [24]. The isotherm study explored the equilibrium relationship between lead and cadmium concentrations in water and their adsorption onto the nanocomposite. By using isotherm models like Langmuir, Freundlich, or BET, researchers analyzed the data to determine adsorption parameters, such as capacity and affinity. These models shed light on surface interactions and whether adsorption occurs in monolayers or multilayers [63]. The sorption of RhB onto a predetermined amount of hybrid hydrogel was examined at different starting concentrations (Ci) in water at 293 ± 275 K and pH 7. Fig. 7a's graph of qe verses Ce (residual RhB in solution at equilibrium) has a convex shape and initially displays a linear sharp rise in qe followed by a gradual decline until saturation point, beyond which there is no increase because RhB molecules have attached themselves to all of the sorption sites of the hybrid material. The highest concentration of RhB sorbed was found in the plot (qm = 1250 mgg^−1^), which was much greater than the concentration found in other analogous hybrid hydrogels of the same size that are listed in Table 3. To further study the sorption isotherm of RhB onto the hybrid hydrogel, the following data was added to the Freundlich (Eq. (7)) isotherm model.

(7) lnqe = lnKF + ln Ce n

where the equilibrium sorbed MG concentration and the corresponding residual concentration in solution are denoted by qe (mg g) and Ce (mol L), respectively. Additionally, the Freundlich parameters KF (mg g-1) and n given in Table 4 were derived using the slope and intercept of the linear trends exhibited in Fig. 7b for lnqe vs lnCe. The hybrid hydrogel's efficient and corporative RhB sorption is demonstrated by the n value (1.872) 1 [15]. The Langmuir adsorption isotherm presented in Eq. (8) below also included the RhB-sorption data;

(8) Ce qe = 1 KLqm + Ce qm

**Table 3:** Comparison of the maximal sorption capacity between our study and previous research.

Sorbent used	q_m_ (max) (mg/g)	sources
Polyacrylic acid hydrogel	1123	[64]
Guar-gum and acrylic acid	61.92	[65]
Locust bean gum based hydrogel	142.85	[66]
Gellan gum based hydrogel	45.45	[67]
Gellan gum/bacterial cellulose hydrogel	17.57	[68]
Gellan gum based hydrogel for removal of cationic dyes	552.48	[69]
Hydrogel based on starch graft acrylic acid	260	[70]
Gum gatti and acrylic acid based hydrogel	909.9	[71]
acrylic acid-grafted sodium alginate-based TiO2 hydrogel nanocomposite	1156.61	[72]
Present hydrogel based on gellan gum/acrylic acid	1250

**Table 4:** The acquired varied parameters for RhB-sorption onto p(GG-co-AAc) hydrogel from Freundlich, Langmuir, and Temkin isotherm models.

Isotherms	Freundlich			Langmuir				Temkin		
Parameters	**K**_**F**_**(mg g**^**−**^**^1^)**	$\frac{1}{n}$**(L g**^**−**^**^1^)**	**R**^**2**^	**q**_**m**_**(mg g**^**−**^**^1^) Exp**	**q**_**m**_**(mg g**^**−**^**^1^)** **Cal**	$K_{L}$**(L g**^**−**^**^1^)**	**R**^**2**^	**b (j mol**^**−**^**^1^** **K**^**−**^**^1^)**	$K_{T}$**(**${mg\;g}^{-1})$	**R**^**2**^
293K	338.84	0.2632	0.5633	1174.402	1250	0.171	0.9989	177.64	5.794	0.6963

The slope and intercept of the Ce/qe vs. Ce linear segments presented in Fig. 7c were used to compute the maximal sorption potential (qm, expressed in mg g-1) and the Langmuir constant (KL, expressed in L g^−1^). The separation factor (RL), which is crucial for predicting whether sorption would be favorable, unfavorable, or irreversible, was calculated using Eq. (9) and the Kl value.

(9) RL = 1 1 + KLCo

The RL values (0.386− 0.134)< 1 indicate that the hybrid hydrogel's RhB sorption is of a favorable nature [63]. A Temkin isotherm was also applied to the sorption data, as shown below in Eq. (10)

(10) Cq = RT b lnKT + RT b lnCe

The quantities of adsorbed RhB, the gas variable, and the temperature in Kelvin (K) are all represented by the letters Cq, T, and R in the Temkin equation (Eq. (10)). The slope and intercept of the Temkin plot seen in Fig. 7d were used to determine the Temkin coefficient (KT) and b (describe sorption enthalpy) values, which are reported in Table 3. Based on the R^2^ value, the data is consistent with the Langmuir, Temkin, and Freundlich isotherm models. The more accurate outcomes that conform to the Langmuir model demonstrate that RhB was chemisorbed onto the hydrogel composite as a result of an electrostatic contact between both the cationic RhB and the anionic sites (COO). On the other hand, the Freundlich isotherm demonstrated the least fit as seen by the comparably low R^2^ value, which may rule out the likelihood of physisorption and multilayer growth during sorption.

#### 3.4.3 Thermodynamics sorption study

The thermodynamics of adsorption can be described using concepts such as Gibbs free energy, enthalpy, and entropy. Gibbs free energy is a measure of the available energy in a system that can be used to do work. Enthalpy is the heat energy absorbed or released during a reaction, while entropy is a measure of the disorder or randomness in a system.

**Fig. 8:**
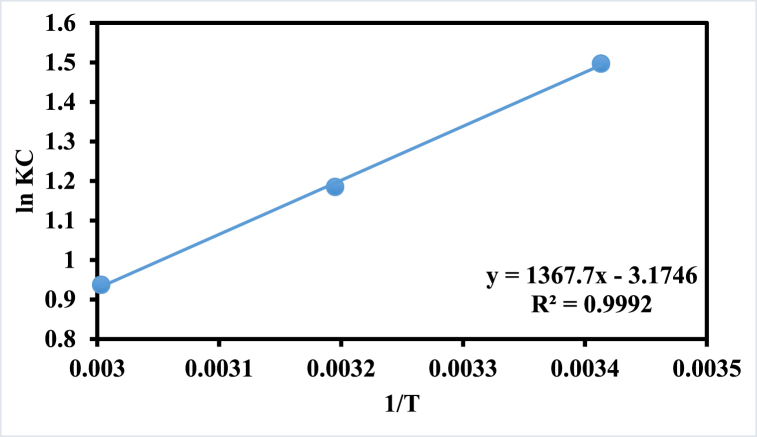
Thermodynamics study of p(GG-co-AAc) hybrid hydrogel.

The adsorption process can be classified into two types: physical adsorption and chemical adsorption. Physical adsorption involves weak forces such as van der Waals forces, while chemical adsorption involves stronger forces such as covalent bonding. Negative enthalpy values are often observed in the process of adsorption, particularly in exothermic adsorption reactions. This is because the adsorption process involves a transfer of heat energy between the adsorbent (the surface onto which adsorption occurs) and the adsorbate (the substance being adsorbed). The entropy is negative to show that the system is in less disorder. The values of thermodynamics parameters have been estimated from graph given in Fig. 8 whereas their values are summarized in Table 5.

**Table 5:** The acquired varied parameters for RhB-sorption onto p(GG-co-AAc) hydrogel from thermodynamic study.

ΔG° Jmol^−1^			ΔH° KJmol^−1^	ΔS° J mol^−1^K^−1^
293 K	313 K	333 K	−11371	−26.39
−3639	−3111	−2584		

#### 3.4.4 pH effect on sorption

**Fig. 9:**
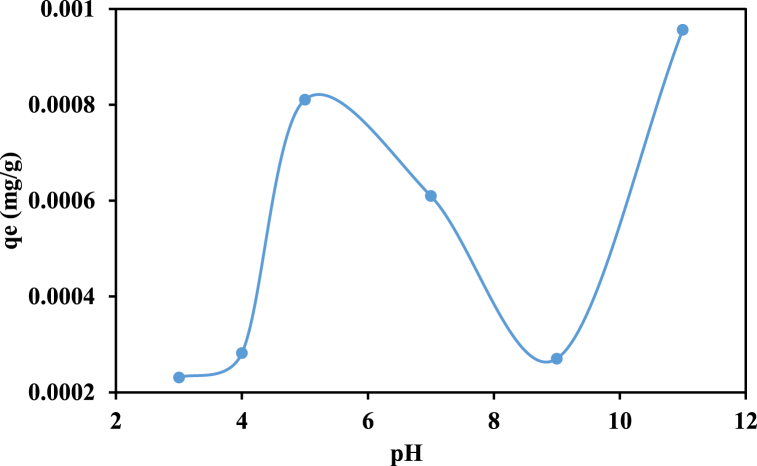
Investigation of pH effect on RhB sorption capacity of p(GG-co-AAc) hybrid hydrogel.

The hybrid sorbent's capacity to eliminate RhB (qe) changed erratically in reaction to the pH of the aquatic environment, as seen in Fig. 9. RhB sorption is low at pH 3, rises noticeably to pH 5, drops off steadily until pH 9, and then rises dramatically from pH 9 to 11. The oddity may be explained by the structural alterations that RhB and hybrid hydrogel underwent in both alkaline and acidic environments. Since the dye is now more positively charged, the anionic hydrogel may attach to it more effectively as a result. On the other hand, the p(GG-co-AAc) hydrogel becomes more negatively charged in an alkaline environment when the pH rises due to an increase in the degree of deprotonation [73]. As a result, in the current case, sorption is monitored by the hybrid material's equilibrium between RhB protonation and –COOH deprotonation. The substantial levels of protonated RhB and deprotonated sorbent, both of which are easily electrostatically bound, may be the reason for the pH 5 maximum sorption potential.

#### 3.4.5 Recycling performance

**Fig. 10:**
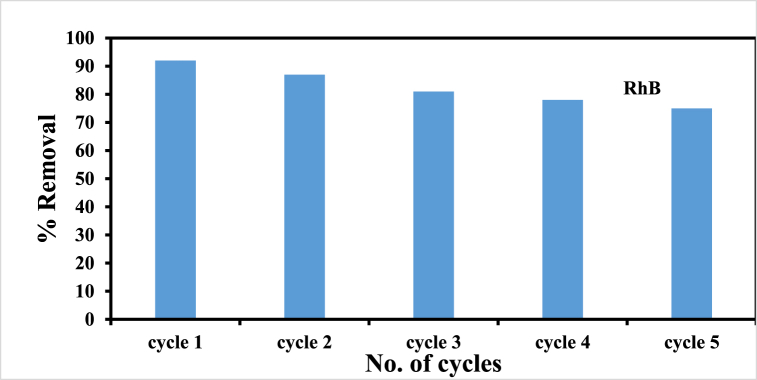
Recycling behavior of p(GG-co-AAc) hybrid hydrogel.

In terms of simple regeneration by one-step desorption of trapped dyes, hydrogels are better sorbents than conventional materials. Using a solvent extraction procedure, the RhB-loaded hybrid sorbent was effectively recovered by dissolving the RhB molecules trapped inside the composite material in acetone (10 mL). Five different sorption-desorption processes were used to examine the potential for recycling the sorbent shown in Fig. 10. As a consequence of the recycling process, the hybrid material experienced a 7% decrease in the amount of Rhodamine B (RhB) removed compared to the initial run, primarily due to the dyes detaching from the hydrogels. The results have been presented in Fig. 10.

## 4 Conclusions

The FTIR, XRD, and EDX results supported the creation of an unique poly(Gellan gum-co-acrylic acid) hydrogel. The composite surface was extremely granular and rough, as shown in the SEM micrographs, which made it the ideal surface for the sorption of dangerous dyes. After 23 h, the superabsorbent reached swelling equilibrium in distilled water, and when the pH of the water rose, so did the degree of swelling. The sorption kinetic data showed good agreement only with pseudo-second order model, as seen by higher R^2^ values and a significant agreement among experimental and theoretical qe values.The Langmuir isotherm model seems to better suit the sorption data, according to the plot's highest R^2^ value. According to pseudo-second order kinetic and Langmuir isotherm models, RhB was physically taken onto the hydrogel as a response of the electrostatic bond formation between cationic dye and anionic sorbent. a significant tendency for swelling (3230%) and sorption (1250 mgg^−1^).

## Author contribution statement

Salma Jabeen: Sultan Alam: Luqman Ali Shah:Muhammad Zahoor: Conceived and designed the experiments; Performed the experiments; Analyzed and interpreted the data; Wrote the paper. Muhammad Naveed Umar: Riaz Ullah: Conceived and designed the experiments. Salma Jabeen: Muhammad Zahoor: Performed the experiments. Muhammad Naveed Umar: Riaz Ullah:Analyzed and interpreted the data; Muhammad Naveed Umar: Riaz Ullah: Contributed reagents, materials, analysis tools or data.

## Data availability statement

Data will be made available on request.

## Funding statement

Researchers supporting Project Number (RSP2023R110) 10.13039/501100002383King Saud University, Riyadh, Saudi Arabia.

## Declaration of competing interest

The authors declare that they have no known competing financial interests or personal relationships that could have appeared to influence the work reported in this paper.
